# Alterations in *LMTK2*, *MSMB *and *HNF1B *gene expression are associated with the development of prostate cancer

**DOI:** 10.1186/1471-2407-10-315

**Published:** 2010-06-22

**Authors:** Lorna W Harries, John RB Perry, Paul McCullagh, Malcolm Crundwell

**Affiliations:** 1 Institute of Biomedical and Clinical Sciences, Peninsula NIHR Clinical Research Facility, University of Exeter, Peninsula Medical School, Barrack Road, Exeter, Devon, UK; 2 Genetics of Complex Traits, Peninsula Medical School, University of Exeter, Exeter, UK; 3 Department of Histopathology, Royal Devon and Exeter NHS Foundation Trust, Exeter, UK; 4 Department of Urology, Royal Devon and Exeter NHS Foundation Trust, Exeter, UK

## Abstract

**Background:**

Genome wide association studies (GWAS) have identified several genetic variants that are associated with prostate cancer. Most of these variants, like other GWAS association signals, are located in non-coding regions of potential candidate genes, and thus could act at the level of the mRNA transcript.

**Methods:**

We measured the expression and isoform usage of seven prostate cancer candidate genes in benign and malignant prostate by real-time PCR, and correlated these factors with cancer status and genotype at the GWAS risk variants.

**Results:**

We determined that levels of *LMTK2 *transcripts in prostate adenocarcinomas were only 32% of those in benign tissues (p = 3.2 × 10^-7^), and that an independent effect of genotype at variant rs6465657 on *LMTK2 *expression in benign (n = 39) and malignant tissues (n = 21) was also evident (P = 0.002). We also identified that whilst *HNF1B(C) *and *MSMB2 *comprised the predominant isoforms in benign tissues (90% and 98% of total *HNF1B *or *MSMB *expression)*, HNF1B(B) and MSMB1 *were predominant in malignant tissue (95% and 96% of total *HNF1B *or *MSMB *expression; P = 1.7 × 10^-7 ^and 4 × 10^-4 ^respectively), indicating major shifts in isoform usage.

**Conclusions:**

Our results indicate that the amount or nature of mRNA transcripts expressed from the *LMTK2*, *HNF1B *and *MSMB *candidate genes is altered in prostate cancer, and provides further evidence for a role for these genes in this disorder. The alterations in isoform usage we detect highlights the potential importance of alternative mRNA processing and moderation of mRNA stability as potentially important disease mechanisms.

## Background

Cancer of the prostate is the most common male malignancy in the Western world, accounting for 25% of all male UK cancers (taken from http://info.cancerresearchuk.org/cancerstats). Although survival rates are increasing, cancer of the prostate remains the second most common cause of cancer death in UK men after lung cancer. Several risk factors have been identified, the main factors being age [1], family history [2] and ethnic origin [3]. Prostate cancer is uncommon in men under 50 years old, but 80% of men aged over 80 years were found to have cancerous cells in their prostate at the time of death [1]. Estimates suggest that between 30 - 40% of all early onset cases of prostate cancer (< 55yrs) are caused by inherited factors [2], highlighting the importance of genetics in this disorder. Early-onset prostate cancer that aggregates in families is likely to result from the inheritance of rare genetic variants that have a large impact. Several strong risk factors have been identified from family studies which include mutations in the *ELAC2*, *RNASEL *and *MSR1 *genes involved in immune response [4] and the *BRCA1 *and *BRCA2 *genes involved in the development of breast cancer [5].

Despite increases in the understanding of the familial forms of prostate cancer, the aetiology of the remaining 60% of cases remains unclear. The advent of genome wide association studies (GWAS) has resulted in a dramatic increase in the number of susceptibility loci that have been associated with the development of prostate cancer (table 1). Four independent studies in the Icelandic, UK and American populations have identified more than 20 distinct genomic locations as being implicated in susceptibility to prostate cancer [6-9].

**Table 1:** Loci that have been identified as significant determinants of prostate cancer risk in four independent genome wide association studies.

Gene	Location	Marker	MAF	Post replication P value	Position within gene	Source
*HNF1B*	17q21.3	rs4430796 rs11649743	0.479 0.479	9.58 × 10-^10^ 1.7 × 10^-9^	IVS2 IVS4	[7,8,34]

*MSMB*	10q11.2	rs10993994	0.339	8.7 × 10^-29^ 7.31 × 10^-13^	5' UTR	[8,9]

*LMTK2*	17q25.3	rs6465657	0.438	1.1 × 10^-9^	IVS9	[9]

*SLC22A3*	6q26-q27	rs9364554	0.233	5.5 × 10^-10^	IVS5	[9]

*CTBP2*	10q26.13	rs4962416	0.229	1.70 × 10^-7^	IVS1	[8]

*MYEOV*	11q13	rs10896449	0.492	1.76 × 10^-9^	Intergenic, 5' to gene	[8]

*JAZF1*	7p15.2-p15.1	rs10486567	0.267	2.14 × 10^-6^	IVS2	[8]

MAF = minor allele frequency in the HapMap CEU population.

Many of the GWAS association signals identified are located in regions of the genome that are not translated into protein. This may indicate that the associated marker is in close linkage disequilibrium with an uncharacterised variant in the protein coding region, or that the functional SNP may cause its effect by epigenetic or histone modification. The other possibility is that the variant may act at the level of the messenger RNA.

There are several mechanisms by which variants in non-coding regions of the mRNA transcripts could affect gene function. Firstly, if the variant lies in the promoter or 5' untranslated region of the transcript, it may interfere with the efficiency of transcription or translation, as has been shown to be the case for several human and rodent genes [10-12]. Variants located within the intronic sequences may interfere with mRNA splicing [13]. The production of aberrant splice products has previously been shown to be very important in the aetiology of cancer in general [14,15], but may contribute to the biological heterogeneity of prostate cancer in particular [16]. Finally, polymorphisms located in the 3' untranslated region may disrupt regulatory elements necessary for mRNA stability, interfere with regulation of translation by microRNAs or influence the polyadenylation dynamics of the transcript [17-19].

**Figure 1:**
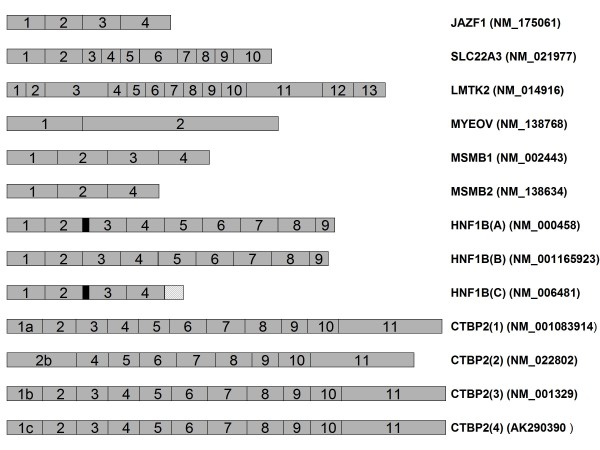
**Schematic representation of the structure of transcripts studied here**. The structure of the *JAZF1*, *SLC22A3*, *LMTK2*, *MYEOV*, *MSMB*, *HNF1B *and *CTBP2 *mRNAs are represented here, together with their RefSeq ID numbers. Exons are represented by numbered grey boxes. The black box represents the section of exon 3 absent from isoform *HNF1B(B)*. The section of intronic sequence present in *HNF1B(C) *is indicated by a hatched box.

In this series of experiments, we sought to investigate whether the genes suggested by the GWAS for prostate cancer were differentially expressed in malignant prostate tissues, and whether their expression was influenced by genotype at the GWAS risk variants. Seven genes were selected for study (figure 1). We measured the total or isoform specific gene expression of the *JAZF1 (*JAZF zinc finger 1), *CTBP2 (*C-terminal binding protein 2), *LMTK2 (*lemur tyrosine kinase 2), *SLC22A3 (*solute carrier family 22 extraneuronal monoamine transporter, member 3), *MYEOV (*myeloma overexpressed), *MSMB *(microseminoprotein beta) and *HNF1B *(HNF homeobox 1 beta) in a cohort of 39 non-malignant benign prostatic hyperplasia (BPH) samples and 21 prostate adenocarcinoma samples, and correlated gene expression with cancer status and with the genetic variants identified in the GWAS (table 1). We demonstrate two of the eight variants are correlated with gene expression levels in non-malignant prostate tissues, and that the presence of adenocarcinoma is associated with altered gene expression levels and differential expression of alternatively processed isoforms for three of the candidate genes in prostate tissue itself.

## Methods

### Subject Details

The prostate samples in this study were obtained from the Exeter tissue bank, and comprised prostate chippings taken during routine transurethral resection of the prostate (TURP). Both cancer and BPH groups originated from a population of older men treated for urological problems at the Royal Devon and Exeter Hospital, Exeter, UK and were predominantly of Caucasian origin. Samples were flash frozen in liquid nitrogen upon removal, and stored at -80°C until required. Subjects with benign prostatic hyperplasia (n = 39) had no sign of prostate malignancy at the time the samples were taken, and none had received a subsequent diagnosis of prostate cancer 5 years following their diagnosis of benign prostatic hyperplasia (BPH). The median age of the BPH cohort was 83 years (IQR 78,88) and the median age of the prostate cancer cohort was 83 years (IQR 76,87). BPH samples were used as the control group for several reasons; firstly, most males have evidence of BPH by the age of 70 or 80, so the presence of some degree of BPH is 'normal' at the median age at diagnosis in our prostate cancer cohort (77 yrs, [IQR 72,82]). Truly 'normal' prostate tissue would thus only be obtained in a much younger control cohort, which could introduce bias. Secondly, since collection of prostate tissue requires a surgical procedure, which would only be undertaken in subjects with evidence of symptoms of prostate enlargement. Even then, morphologically normal tissue may harbour premalignant genetic abnormalities that are not immediately obvious. However, we acknowledge that genetic heterogeneity between patients and controls may influence the gene expression from individual to individual. Prostate cancer samples (n = 21) underwent confirmation of cancer status, histology and Gleason scoring by dissection of the TURP chips and subsequent histological examination of tissue from either side of the sample used for RNA analysis. All cancer samples were classified as prostatic adenocarcinomas, of which 57% were Gleason score 6 or 7, and 43% were Gleason score 8-10. Further analysis of patient case notes revealed that 8/19 prostate cancer patients and 6/37 Benign Prostatic Hyperplasia patients had been exposed to hormone treatment (Zoladex, Finasteride, Casodex or Proscar).

### Ethics Statement

All participants had consented to their tissue being used in this study, and ethical approval was obtained from the South West Research Ethics Committee. This study was carried out according to the Declaration of Helsinki.

### DNA extraction

DNA was extracted from prostate chippings using the Qiagen DNA mini kit (Qiagen, Crawley, UK) using standard procedures on the fully automated QiaCube sample preparation platform (Qiagen, Crawley, UK), following manual homogenisation of tissue.

### Choice of candidate genes

Seven candidate genes were identified for expression analysis. These were chosen on several criteria. Firstly, we chose our candidate genes on the basis that the GWAS index SNPs were located within the gene sequence itself, or very close to the genes in question. The linkage disequilibrium blocks identified in the GWAS may contain other genes (or variants) that may contribute to the risk of developing prostate cancer, the majority of these are located remotely to the association signal and do not represent better biological candidates. Secondly, all seven are good biological candidates, having roles in cell proliferation, regulation of apoptosis or prostate development and function. Analysis of the linkage disequilibrium structure around the GWAS index SNPs revealed several other candidates, but these were not analysed due to a lack of expression in the prostate, a lack of convincing biological evidence for a role in prostate function or the presence of more plausible candidate genes. The remaining four signals located on chromosomes 3, 8, 19 and X were not analysed due to the lack of an obvious candidate gene.

### Genotyping

Genotyping was carried out by PCR amplification and direct sequencing of the sequence flanking the variant in question; primers and conditions are available on request. Amplification reactions contained 10 μl Megamix Royal (Microzone, Haywards Heath, UK), 10 μM primers and 50 ng DNA in a total volume of 20 μl. Sequence specific primers for each amplicon were tagged with 5' M13 tails to allow sequencing to be performed with a universal M13 primer. Single-strand sequencing was carried out using standard methods on an ABI 3730 (Applied Biosystems, Warrington, UK). Sequences were compared to a designated individual homozygous for the major allele of the variant using Mutation Surveyor (version 3.20; SoftGenetics, State College, PA).

### RNA extraction

RNA was extracted from prostate chippings using the Qiagen RNAeasy kit (Qiagen, Crawley, UK) on the fully automated QiaCube sample preparation platform (Qiagen, Crawley, UK), following manual homogenisation of tissue with an additional pass through a Qiagen Qiashreddder column (Qiagen, Crawley, UK) column to shear genomic DNA. RNA samples were stored at -80°C prior to analysis.

### Reverse Transcription

Approximately 500 ng of mRNA from each sample was treated with 1u of RNAse-free DNAse (Promega, Madison, USA) for 30 minutes at 37°C followed by 65°C for 10 minutes for nuclease inactivation. Complementary DNA (cDNA) was synthesized from mRNA using the Superscript III VILO RT-PCR system (Invitrogen, Paisley, UK) according to manufacturer's instructions, with an incubation temperature of 42°C and a reaction volume of 40 μl.

### Quantitative real time PCR for the measurement of total or isoform-specific gene expression

Real-time PCR reactions using target-specific probes were carried out in triplicate using the ABI Prism 7900 HT platform (Applied Biosystems, Warrington, UK) and contained 5 μl TaqMan Fast Universal Mastermix (no AMPerase) (Applied Biosystems, Warrington, UK), 0.9 μM each primer, 0.25 μM probe and 2 μl cDNA reverse transcribed as above in a total volume of 10 μl. PCR conditions were a single cycle of 95°C for 20 seconds followed by 50 cycles of 95°C for 1 second and 60°C for 20 seconds. Individual probe and primer sets were pre-validated off-the-shelf assays to specific transcripts provided by Applied Biosystems (Warrington, UK; Assay identification numbers available on request). The relative expression level of each isoform was then determined relative to the *B2 M *and *GUSB *transcripts by the comparative (ΔΔCt) method [20]. The abundance of each target in each individual was normalized to the average measurement for the transcript in question across the cohort prior to correlation of expression levels with genotype.

### LMTK2 Allele-specific expression

Measurement of the relative abundance of transcripts bearing different alleles at variant rs3801294, in perfect linkage disequilibrium (r^2 ^= 1; D' = 1) with the *LMTK2 *index SNP rs6465657, was carried out in single tube reactions by allele-specific real time PCR. PCR amplicons were generated by a common set of primers for both alleles, but transcripts bearing different alleles were discriminated by the use of allele-specific probes labelled with different fluorochromes (6-fluorescein [6-FAM] and VIC); probes and primers available on request. This allows a measurement of transcript abundance that is independent of amplification efficiency. Real-time PCR reactions using were carried out in triplicate using the ABI Prism 7900 HT platform (Applied Biosystems, Warrington, UK) and contained 5 μl TaqMan Fast PCR Master Mix, no AmpErase™ (Applied Biosystems, Warrington, UK), 72 μM each primer and 16 μM each probe) in a total volume of 10 μl. PCR conditions were a single cycle of 95°C for 20 seconds followed by 60 cycles of 95°C for 1 second and 60°C for 20 seconds. The expression level of transcripts bearing the G allele (marking the C allele at rs6465657) was then calculated relative to that of transcripts bearing the A allele (marking the T allele at rs6465657) by the comparative (ΔΔCt) method [20], as we have done for several previous studies [21,22]. Quantifications were then compared to the average measurement obtained from eleven independent DNA samples, which should provide a known 50:50 mix of A and G-bearing transcripts, in order to control for differences in probing efficiencies.

### Statistics

The statistical significance of apparent differences in gene expression levels was investigated by Kruskal Wallis analysis for multiway comparisons and by Mann Whitney-U analysis for pairwise comparisons. Non-parametric statistics were employed due to the relatively small sample numbers and the fact that the data obtained were not normally distributed. In the case of the association between *LMTK2 *expression and cancer status, linear regression was carried out on natural logarithmically transformed data to allow adjustment for the effect of genotype at rs6465657, which we found to be independently associated with *LMTK2 *expression. All statistical analyses were carried out using SPSS v15.0 (SSPS PLC, Chicago, USA).

## Results

### Genotyping of GWAS risk loci in benign prostatic hyperplasia and prostate cancer samples

We obtained 426/480 genotypes for the 8 GWAS variants in our cohort of 39 benign prostatic hyperplasia specimens and 21 prostate adenocarcinoma samples (310 for the BPH cohort and 116 for the cancer cohort). All three genotypes were detected for the variants, with the exception of minor allele (C) homozygotes for rs4962416 within the *CTBP2 *gene, as expected given the minor allele frequency of only 0.229 in the HapMap CEU population.

### Risk genotypes at rs6465657 and rs4962416 may be associated with differences in LMTK2 and CTBP2 expression levels in benign prostatic hyperplasia samples

Analysis of potential correlations between genotype at the GWAS association loci and isoform-specific gene expression of nearby candidate genes revealed correlations between risk genotype at the GWAS variants rs6465657 and rs4962416 with the expression of *LMTK2 *and *CTBP2 *respectively. Individuals carrying two copies of the protective T allele of rs6465657 expressed almost a third less *LMTK2 *mRNA than did individuals carrying one or two copies of the risk 'C' allele (0.68 [Interquartile range [IQR] = 0.63] v/s 1.18 [IQR = 0.56]; P = 0.007). Similarly, individuals homozygous for the 'T' allele at rs4962416 expressed 37% less of a partially characterised isoform of *CTBP2*; *CTBP2(4)*, than did individuals with CT genotypes (0.82 [IQR 0.83] v/s 1.31 [IQR 0.96]; P = 0.009). Although neither of these findings quite reaches full statistical significance following adjustment for multiple testing (P = < 0.004 for 12 independent tests), they do indicate that these genes are worthy of further study.

### Allele-specific differences in the expression of LMTK2 transcripts bearing different alleles of rs6465657

**Figure 2:**
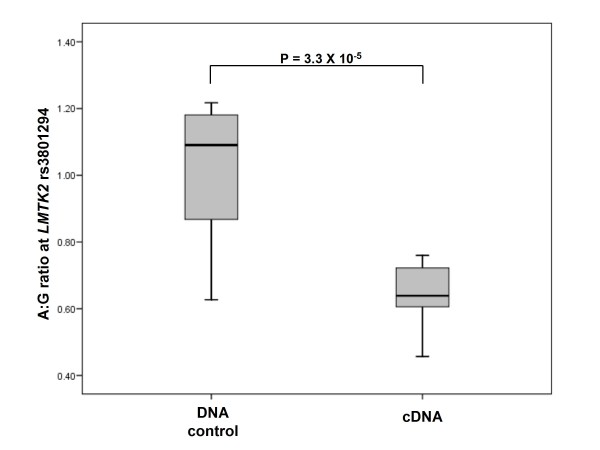
**Allele-specific expression of *LMTK2 *transcripts**. This histogram demonstrates allele-specific differences in the expression of *LMTK2 *transcripts bearing either the G or A allele at rs3801294, which is in perfect linkage disequilibrium (r^2 ^= 1; D' = 1) with the index SNP rs6465657. The sample identity is given on the X-axis and the ratio of transcripts carrying G alleles (marking the C allele of rs6465657) relative to those carrying the A allele (marking the T allele at rs6465657) is given on the Y-axis. Error bars represent the interquartile range (IQR) of the measurements. The level of significance as determined by pairwise Mann Whitney-U analyses is indicated by asterisks.

We measured the abundance of each *LMTK2 *allele of rs6465657 in 17 heterozygous individuals, and determined that transcripts bearing the rs3801294 'A' allele, which is in perfect linkage disequilibrium (r^2 ^= 1; D' = 1) with the protective 'T' allele at rs6465657, were significantly less abundant in heterozygous BPH tissues than were those carrying the 'G' allele, linked to the 'C' allele of rs6465657. The ratio of protective 'A' alleles to risk 'G' alleles in heterozygous individuals is 0.65 [IQR 0.12]:1 [IQR 0.33]; P = 3.3 × 10^-5^, figure 2), which remains statistically significant after adjustment for multiple testing. This provides further evidence for a role of *LMTK2 *in determining susceptibility to prostate cancer. No correlations of genotype with expression levels were noted for the remaining isoforms of the *CTBP2 *gene, or any isoform of the *JAZF1*, *MYEOV*, *SLC22A3*, *HNF1B *or *MSMB *genes in BPH tissues.

### Total LMTK2 expression is altered in prostate cancer tissues compared with non-malignant BPH samples

**Figure 3:**
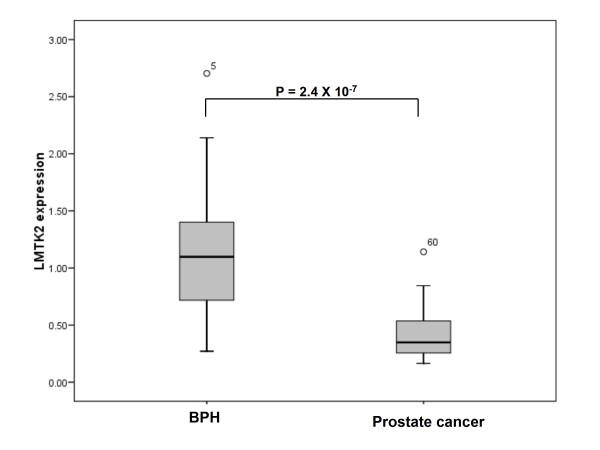
**Expression of *LMTK2 *transcripts is reduced in prostate cancer**. This boxplot gives the cancer status (benign prostatic hyperplasia or prostate adenocarcinoma) on the X-axis, and the expression of the single isoform of the *LMTK2 *gene relative to the endogenous controls beta-2-microglobulin (*B2M*) and beta-glucorinidase (*GUSB*) on the Y-axis. Error bars represent the interquartile range (IQR) of the measurements. The level of significance as determined by linear regression of natural logarithmically transformed data is indicated. Outliers are indicated by circles.

We correlated expression levels at each of the loci with prostate cancer status (table 2). We found that expression levels of the *LMTK2 *gene were also inversely correlated with the presence of prostate cancer. Tumour samples expressed 68% less *LMTK2 *mRNA than did non-malignant BPH samples (figure 3; relative expression levels were 1.10 [IQR 0.77] in BPH versus 0.35 [IQR 0.30] in prostate cancer; p = 2.4 × 10^-7^), after correction for the potential effect of genotype at rs6465657. Amongst the eight members of the prostate cancer cohort who had exposure to antiandrogenic drugs, no effect of prior hormone treatment on the expression levels of *LMTK2 *was noted (0.49 [IQR 0.29) for untreated subjects versus 0.32 [IQR 0.30] in treated subjects; P = 0.270).

**Table 2:** Total expression levels of candidate genes in non-malignant and prostate cancer tissues.

Gene	Total Expression in non-malignant prostate tissues	Total Expression in prostate cancer tissues	Value
JAZF1	0.82 (1.7)	0.83 (0.97)	0.185

SLC22A3	1.01 (1.42)	0.48 (0.75)	0.015

***LMTK2***	***1.10 (0.77)***	***0.35 (0.30)***	***2.4 × 10^-7^***

MSMB	95.3 (326.3)	13.5 (90.9)	0.156

HNF1B	63.0 (106.8)	37.3 (56.0)	0.175

MYEOV	0.07 (0.15)	0.08 (0.15)	0.655

CTBP2	3.35 (2.54)	2.1 (1.6)	0.008

Gene expression levels are analysed relative to the beta 2 microglobulin (*B2M*) and beta glucorinidase (*GUSB*) genes, and normalised to the average value in the non-malignant samples. The interquartile range is given in parentheses. A P value of 0.007 (P = 0.05/n° of tests) was taken to indicate statistical significance following correction for multiple testing (7 independent tests). Statistically significant results are indicated in bold italic type.

We also noted differences in the total expression of the *SLC22A3 and CTBP2 *genes in BPH compared with prostate cancer. *CTBP2 *and *SLC22A3 *mRNA levels were reduced by 27% and 50% in prostate cancer tissues compared with BPH tissues (4.2 [IQR 2.5] v/s 2.1[IQR 1.6]; P = 0.008 for *CTBP2 *and 0.48 [IQR 0.75] v/s 1.01 [IQR 1.42]; P = 0.015 for *SLC22A3*). *CTBP2(1) *and *CTBP2(4) *transcripts showed a trend towards lower expression in prostate cancer subjects treated with hormone therapy (*CTBP2(1) *; (0.87 [0.40] in untreated subjects versus 0.31[IQR0.17]; p = 0.006) and *CTBP2(4) *transcripts (1.01[IQR 0.99] in untreated subjects versus 0.45 [IQR0.42] in treated subjects; p = 0.006) but these results do not reach statistical significance following adjustment for multiple testing (P = < 0.004 for 12 independent tests). This observation was also not noted in the BPH cohort (P = 0.77 and 0.69 for *CTBP2(1) *and *CTBP2(4) *respectively. No association of cancer status with expression level was found for a control gene (SFRS protein kinase 1; *SRPK1*), not known to be associated with prostate cancer in any GWAS. We did not identify any correlations of total gene expression with cancer status for the *JAZF1*, *MSMB*, *HNF1B*, *MYEOV *or *CTBP2 *genes. No correlations with transcript expression were noted for cancer stage (assessed by Gleason grading of the cancer) or with exposure to antiandrogenic drugs for any other transcripts analysed in either prostate cancer or BPH cohorts.

### Alterations to the relative balance of alternatively spliced forms of the MSMB and HNF1B genes in prostate cancer tissues

**Figure 4:**
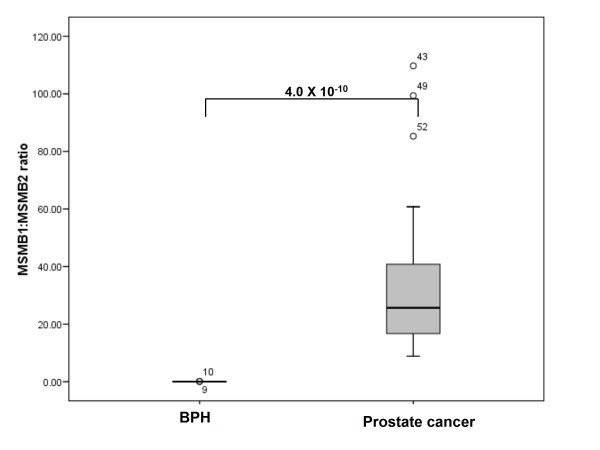
**The ratio of alternatively-expressed isoforms of the *MSMB *gene is altered in prostate cancer tissues**. This boxplot gives the cancer status (benign prostatic hyperplasia or prostate adenocarcinoma) on the X-axis, and the ratio of *MSMB1:MSMB2 *isoform expression calculated relative to endogenous controls beta-2-microglobulin (*B2M*) and beta-glucorinidase (*GUSB*) on the Y-axis. Error bars represent the interquartile range (IQR) of the measurements. The level of significance as determined by pairwise Mann Whitney-U analyses is indicated. Outliers are indicated by circles.

**Figure 5:**
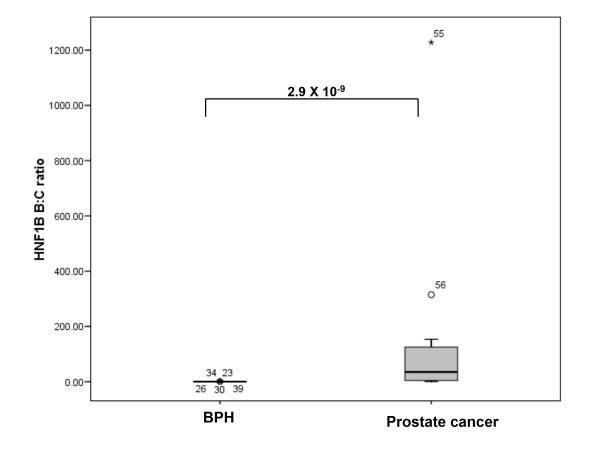
**The ratio of alternatively-expressed isoforms of the *HNF1B *gene is altered in prostate cancer tissues**. This boxplot gives the cancer status (benign prostatic hyperplasia or prostate adenocarcinoma) on the X-axis, and the ratio of *HNF1B(B):HNF1B(C) *isoform expression calculated relative to endogenous controls beta-2-microglobulin (*B2M*) and beta-glucorinidase (*GUSB*) on the Y-axis. Error bars represent the interquartile range (IQR) of the measurements. The level of significance as determined by pairwise Mann Whitney-U analyses is indicated. Outliers are indicated by circles.

Although we found no differences in total *HNF1B, CTBP2 *or *MSMB *expression between BPH and prostate cancer cases, these genes are alternatively processed. We therefore sought to determine if there were differences in the relative balance of isoforms within our sample cohort (table 3). We found highly significant disturbances to the profile of isoforms expressed in prostate cancer in the case of the *MSMB *and *HNF1B *genes. In BPH tissues, the primary isoform expressed at the *MSMB *locus is *MSMB2*, which comprises 98% of *MSMB *expression in this tissue type. However, in cancer tissues, there is a near complete switch to isoform *MSMB1*, which is present at 96% total *MSMB *expression in prostate cancer. The ratio of *MSMB1*:*MSMB2 *isoforms is altered from 0.02 [IQR 0.02]: 1 in BPH tissue to 25.7 [IQR 30.2]: 1 in prostate adenocarcinoma tissues (P = 4.0 × 10^-10^, figure 4). In the case of the *HNF1B *gene, coding for 3 isoforms, we noted a similar switch in isoform expression. The predominant isoforms in BPH samples is *HNF1B(C)*, present at 90% of total *HNF1B *expression. In prostate cancer tissues however, isoform *HNF1B(B) *is predominant at 95% total *HNF1B *expression, with the levels of *HNF1B(C) *dropping to only 3%. The proportion of isoform *HNF1B(A) *does not change. The ratio of *HNF1B(B)*:*HNF1B(C) *isoforms changes from 0.7 [IQR 0.22]: 1 in BPH to 35.4 [IQR 124.71]: 1 in prostate cancer tissues (P = 2.9 × 10^-9^; figure 5). We also found 47% and 50% reductions in *CTBP2(1) *and *CTBP2(4) *expression levels in prostate cancer tissue compared with benign prostate tissues (P = 0.009 and 0.007 for *CTBP2(1) *and *CTBP2(4) *respectively), but these values do not reach statistical significance following adjustment for multiple testing (P = < 0.003 for 12 tests). Previous exposure to antiandrogenic drugs did not affect the ratio of *MSMB *or *HNF1B *splice variants in samples from prostate cancer or BPH patients (P = 0.24 in untreated subjects and 0.69 in treated subjects for prostate cancer cohort and P = 0.88 24 in untreated subjects and 0.72 in treated subjects for the BPH cohort).

**Table 3:** Isoform-specific expression levels of candidate genes in non-malignant and prostate cancer tissues.

Gene	Total Expression in non-malignant prostate tissues	Total Expression in prostate cancer tissues	value
***MSMB1***	***1.8 (6.9)***	***16.2 (85.2)***	***4 × 10^-4^***

***MSMB2***	***125.3 (409.8)***	***0.6 (4.73)***	***5.8 × 10^-5^***

HNF1B(A)	1.2 (2.7)	0.94(1.52)	0.66

***HNF1B(B)***	***5.1 (6.8)***	***35.1 (52.3)***	***1.7 × 10^-7^***

***HNF1B(C)***	***51.8 (109.9)***	***12 (3.4)***	***3.9 × 10^-8^***

CTBP2(1)	1.3 (1.11)	0.62 (0.6)	0.009

CTBP2(3)	0.62 (0.55)	0.45 (0.58)	0.127

CTBP2(4)	1.4 (0.86)	0.70 (0.73)	0.007

Gene expression levels are analysed relative to the beta 2 microglobulin (*B2M*) and beta glucorinidase (*GUSB*) genes, and normalised to the average value in the non-malignant samples. The interquartile range is given in parentheses. A P value of 0.006 (P = 0.05/n° of tests) was taken to indicate statistical significance following correction for multiple testing (8 independent tests). Statistically significant results are indicated in bold italic type.

## Discussion

We report here that disruption of the amount or nature of transcripts expressed from the *LMTK2*, *MSMB *and *HNF1B *genes, identified in the genome wide scans for prostate cancer, may be important in the aetiology of this disorder.

We identified a 68% reduction in the expression of the *LMTK2 *gene in prostate tissue with evidence of the presence of adenocarcinoma when compared with non-malignant BPH samples. Expression levels were 1.10 [IQR 0.77] in BPH versus 0.35 [IQR 0.30] in prostate cancer; p = 3.2 × 10^-7^). We also noted an effect of genotype at the *LMTK2 *variant rs6465657, identified in the GWAS as a susceptibility factor for prostate cancer whereby individuals carrying two alleles of the protective 'T' allele allelic status expressed almost a third less *LMTK2 *than did individuals carrying one or more 'C' alleles (P = 0.002). We suggest that this finding probably arises from a potential imbalance in the transcription or stability of rs6465657 alleles that we note upon allele-specific PCR (figure 1). The precise identity and mode of action of the functional variant tagged by rs6465657 is at present unknown, but It is reasonably easy to postulate that the 'C' allele at rs6465657 could be marking a loss-of-function variant with effects on *LMTK2 *half-life or function.

The *LMTK2 *gene codes for a transmembrane serine/threonine/tyrosine kinase, with a role in endosomal membrane trafficking [23]. It is also associated with NGF-TrkA signalling in murine brain, where it is a negative regulator of NGF-induced neuronal differentiation [24]. LMTK2 interacts negatively with several other proteins with roles in cell division, such as protein phosphatase-1 (*PP1C*) and Inhibitor-2 (*Inh2*), which are part of a complex regulating separation of centrosomes during mitosis [25], and the cyclin-dependent kinase 5 (cdk5)/p35 complex [26], which has several functions, including a role in cell cycle progression. Recent studies have suggested that LMTK2 may interact with myosin IV, which has been shown to regulate both prostate specific antigen (*PSA*) and vascular endothelial growth factor (*VEGF*)[27], both of which are associated with cancer. It is therefore likely that that reduction in the amount or activity of *LMTK2 *may lead to an increase in the proliferative capacity of prostate cells.

We also report a potential role for alternative mRNA processing of the *HNF1B *and *MSMB *genes in the aetiology of prostate cancer. *MSMB *codes for a secreted seminoprotein, which has tumour suppressor properties and is thought to be silenced in prostate tumour tissues by the enhancer of zeste homolog 2 (EZH2) protein [28]. Accordingly, *MSMB *expression has previously been reported to be a positive prognostic indicator in prostate cancer [29], although both these studies measured total, not isoform-specific expression levels. The *MSMB *gene produces 2 isoforms; *MSMB1 *and *MSMB2*, which arise from the skipping of exon 3 in *MSMB2*. This causes a frameshift effect leading to the production of 2 distinct proteins. Previous studies suggest that both *MSMB1 *and *MSMB2 *are present in normal prostate and normal gastric mucosa, but that *MSMB2 *is absent from the majority of a small series of gastric and prostatic carcinomas [30]. This is mostly in agreement with our findings, which suggest a major shift in *MSMB *expression in association with prostate cancer. We found *MSMB2 *to be the predominant isoform in benign prostate tissue, although small amounts (~2%) of *MSMB1 *were also present. In prostate adenocarcinoma tissues, however, *MSMB *expression derived almost completely from *MSMB1*, which is present at 96% of total *MSMB *expression. These findings may indicate that the tumour suppressor properties of *MSMB *are derived from isoform *MSMB2*, and that *MSMB1 *isoforms are potentially pro-carcinogenic. This situation is not uncommon for alternatively spliced genes in cancer; the vascular endothelial growth factor (*VEGF*) gene has previously been shown to code for both pro- and anti- angiogenic isoforms, with different behaviours in tumour tissues [16].

The *HNF1B *gene, which encodes three isoforms, *HNF1B(A)*, *HNF1B(B) *and *HNF1B(C)*, in humans [31], demonstrates a similar alteration to the relative balance of alternatively expressed isoforms associated with cancer of the prostate. The predominant isoform switches from *HNF1B(C)*, which is expressed in non-malignant prostate tissues, *to HNF1B(B) *, which is expressed in prostate adenocarcinoma samples. *HNF1B *is a transcription factor expressed in a limited number of tissues, and has previously been associated with renal and ovarian tumours [32,33] as well as prostate cancer [34]. The *HNF1B *isoforms are known to exhibit differences in function and target specificities; Isoforms *(A) *and *(B) *are transcriptional activators whereas isoform *HNF1B(C) *is a transcriptional repressor [35]. *HNF1B *isoforms have also been reported to activate different targets, for example, the *HNF1B(C) *isoform specifically has been demonstrated to negatively regulate the Glutahione-S-transferase A (*GSTA*) promoter, via a mechanism that involves *Il-1beta *[36]. Given the difference in the transcriptional properties and target specificity of these isoforms, we predict that the alterations to *HNF1B *profile we note may manifest as an alteration to the overall activity of the *HNF1B *gene and/or activation of a variant set of its target genes.

Our study provides good evidence that some of the GWAS associations for prostate cancer may be attributed to mRNA effects, but does have a number of caveats. We have chosen a single gene for analysis in the case of each GWAS variant. The variants may tag a large region containing many genes, some of which may also be good candidates. Other genes in the same linkage disequilibrium (D') blocks, or even further distant, could also be important. This was recently documented for the eye colour trait in humans, where variant rs12913832 located within intron 86 of the *HERC2 *gene exerts its effects not by moderating *HERC2 *activity, but by moderating the efficiency of the promoter of the neighboring *OCA2 *gene, located 21Kbp upstream [37]. We have attempted to ensure we have chosen the correct gene on the basis of proximity and biological function, but other genes may also be affected. Our analysis was also based on comparison of samples from patients with prostate cancer, and samples from patients with non-malignant Benign Prostatic Hyperplasia (BPH), rather than a paired analysis due to the difficulty in obtaining normal prostate tissue from the cancer patients. Our results may thus be influenced by factors both within and between groups, such as ethnic origin or the use of antiandrogenic drugs. No differences in the ethnic makeup of the cancer and control cohorts were noted, with both being almost exclusively of Caucasian origin. Similarly, treatment with antiandrogens had a minimal impact on the expression of any of the transcripts studied; with the exception of *CTBP2(1) *and *CTBP2(4) *transcripts, which showed a trend towards lower expression in samples from prostate cancer patients treated with hormone therapy, although this result was not statistically significant and no correlations of the expression of either transcript with hormone therapy was noted in the BPH cohort. No effect of hormone therapy on *LMTK2 *expression, or on the ratio of *MSMB *or *HNF1B *transcripts was noted.

## Conclusions

In this study, we suggest that the overall mRNA expression level and/or the relative balance of alternatively expressed isoforms of the *LMTK2*, *MSMB *and *HNF1B *genes may be important determinants in the developments of prostate cancer, and demonstrate the importance of alternative mRNA processing mechanisms such as alternative splicing or differential use of polyadenylation sites in gene regulation. Our studies highlight a clear need for RNA studies to complement the genome wide association studies for prostate cancer and other diseases.

## Abbreviations

GWAS: Genome wide association study, SNP: Single nucleotide polymorphism, TURP: Transurethral resection of the prostate, BPH: Benign prostatic hyperplasia, mRNA: Messenger RNA, RTPCR: Reverse transcription polymerase chain reaction, 5' UTR: 5' Untranslated region, 3' UTR: 3' Untranslated region.

## Competing interests

The authors declare that they have no competing interests.

## Authors' contributions

LWH designed and executed the study, analysed results and drafted the manuscript. JRBP advised on polygenic aspects of the study and reviewed the manuscript. MC provided BPH and prostate adenocarcinoma samples and PM carried out the histological examination of tissues to prove malignancy. All authors read and approved the final submission.

## Pre-publication history

The pre-publication history for this paper can be accessed here:

http://www.biomedcentral.com/1471-2407/10/315/prepub
